# Smartphone camera oximetry in an induced hypoxemia study

**DOI:** 10.1038/s41746-022-00665-y

**Published:** 2022-09-19

**Authors:** Jason S. Hoffman, Varun K. Viswanath, Caiwei Tian, Xinyi Ding, Matthew J. Thompson, Eric C. Larson, Shwetak N. Patel, Edward J. Wang

**Affiliations:** 1 Paul G. Allen School of Computer Science and Engineering, University of Washington, Seattle, WA USA; 2 Department of Electrical and Computer Engineering, University of California San Diego, La Jolla, CA USA; 3 The Design Lab, University of California San Diego, La Jolla, CA USA; 4 Department of Computer Science, Southern Methodist University, Dallas, TX USA; 5 Department of Family Medicine, University of Washington, Seattle, WA USA; 6 Department of Electrical and Computer Engineering, University of Washington, Seattle, WA USA

**Keywords:** Diagnostic markers, Diagnosis

## Abstract

Hypoxemia, a medical condition that occurs when the blood is not carrying enough oxygen to adequately supply the tissues, is a leading indicator for dangerous complications of respiratory diseases like asthma, COPD, and COVID-19. While purpose-built pulse oximeters can provide accurate blood-oxygen saturation (SpO_2_) readings that allow for diagnosis of hypoxemia, enabling this capability in unmodified smartphone cameras via a software update could give more people access to important information about their health. Towards this goal, we performed the first clinical development validation on a smartphone camera-based SpO_2_ sensing system using a varied fraction of inspired oxygen (FiO_2_) protocol, creating a clinically relevant validation dataset for solely smartphone-based contact PPG methods on a wider range of SpO_2_ values (70–100%) than prior studies (85–100%). We built a deep learning model using this data to demonstrate an overall MAE = 5.00% SpO_2_ while identifying positive cases of low SpO_2_ < 90% with 81% sensitivity and 79% specificity. We also provide the data in open-source format, so that others may build on this work.

## Introduction

**Fig. 1:**
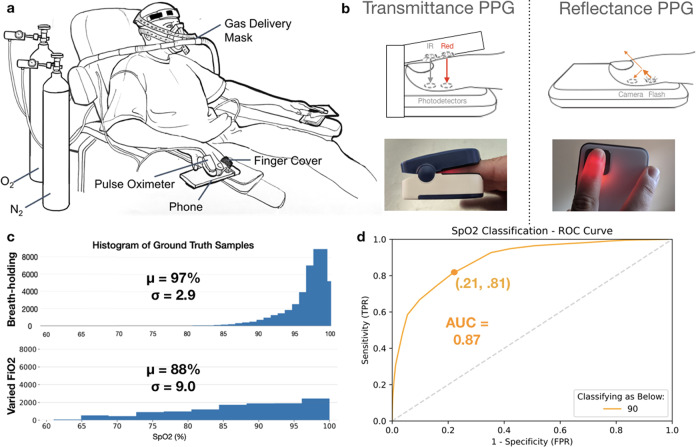
Varied FiO_2_ study using an unmodified smartphone camera. **a** Illustration of the experimental setup of the varied FiO_2_ experiment conducted for this study. The subject breathes a controlled mixture of oxygen and nitrogen to slowly lower the SpO_2_ level over a period of 13–19 min **b** During the study, one finger was placed over a smartphone camera with flash on to record light response via Reflectance PPG, while a second finger was placed in the fingerclip of a tight-tolerance pulse oximeter acting as a transfer standard, which emits Red and IR light reports SpO_2_ via Transmittance PPG. **c** Comparison of the histogram of a breath-holding study dataset, adapted with permission from Ding et al.^3^, with the histogram of the ground truth distribution from our varied FiO_2_ experiment dataset reveal that a more clinically relevant data spread was gathered using this protocol than prior work. **d** Classification results for the smartphone method reveal that 79% of cases of hypoxemia (defined as a low SpO_2_ below 90%) were detected using this method. Illustration and images are the authors’.

Smartphone-based SpO_2_ monitors, especially those that rely only on built-in hardware with no modifications, present an opportunity to detect and monitor respiratory conditions in contexts where pulse oximeters are less available. Smartphone-based solutions for monitoring blood oxygen saturation have been explored previously, employing various solutions used to gather and stabilize the PPG signal^1^, augment the IR-filtered broad-band camera sensor^2^, and filter the resultant signal for noise or outlier correction^3^. Some solutions require extra hardware, such as a color filter or external light source^1,2,4–6^, whereas others rely only on the in-built smartphone hardware and employ software techniques to process the PPG signal^3,7–11^. These prior works indicate that there is potential for smartphone-based SpO_2_ monitors to fill gaps in access to care, but lack validation data on a full range of clinically relevant SpO_2_ levels. Prior evaluation techniques for these smartphone-based studies have been limited to a minimum of 80% SpO_2_ using techniques such as breath-holding, which is limited to short durations of data collection due to participant discomfort, limiting the clinical applicability of the findings. The US Food and Drug Administration (FDA) recommends cleared reflectance pulse oximeter devices achieve <3.5% error across the full range of clinically relevant data of 70–100%^12,13^. To our knowledge, our study is the first to evaluate unmodified smartphone-based pulse oximetry on this range of SpO_2_ data using a Varied Fractional Inspired Oxygen (Varied FiO_2_) study procedure, as shown in (Fig. 1).

Blood-oxygen saturation, reported as SpO_2_ percentage, is one of a number of health measures used by clinicians to assess cardiovascular function, reporting the proportion of hemoglobin in the blood currently carrying oxygen. This ratio can be directly measured from samples of arterial blood using an Arterial Blood Gas (ABG) analysis device. However, obtaining and analyzing arterial blood samples is invasive and can be technically difficult; therefore, it is limited to in-clinic hospital or outpatient lab cases. As a result, clinicians typically rely on the convenience of noninvasive measures of SpO_2_ using FDA-cleared, purpose-built devices called pulse oximeters, consisting of a finger clip and readout screen (Fig. 1b). Pulse oximeters typically perform oxygenation measurement via transmittance photoplethysmography (PPG) sensing at the finger tip, clamping around the end of the finger and transmitting red and IR light via LEDs^14^. By measuring the resultant ratio of light reception on the other side of the finger, the devices leverage the Beer-Lambert Law to estimate the absorption properties of the blood, using calibrated curves based on empirical data to infer blood oxygen saturation^2^. This device allows clinicians to noninvasively monitor SpO_2_ for single (spot-check) or continuous measures.

While baseline SpO_2_ levels vary slightly (typically 96–98% at sea level in otherwise healthy individuals), deviations of 5% or more below these levels can be a sign of more serious cardiopulmonary disease. Respiratory illnesses, such as asthma, chronic obstructive pulmonary disease (COPD), pneumonia, and COVID-19, can cause significant decreases in SpO_2_, hypoxemia (low blood oxygen), and potentially hypoxia (low tissue oxygen). Hypoxia can lead to serious complications, such as organ damage to vital organs like the brain or kidneys, and even death, if uncorrected or occurring acutely for an extended period of time^15^. Repeated measurements of SpO_2_ can be used to monitor for changes in the severity of a wide range of cardiopulmonary conditions such as asthma and COPD^16^, and indicate potential presence of other illnesses including Idiopathic Pulmonary Fibrosis, Congestive Heart Failure, Diabetic Ketoacidosis, and pulmonary embolism^17–19^. Pulse oximetry also has prognostic value; for example, an SpO_2_ level below 90% SpO_2_ has been correlated to increased in-hospital mortality rates for COVID-19 patients^20^ and levels below 95% associated with complications from community-acquired pneumonia^21^ or complications in patients with diagnosed pulmonary embolism^18^. Thus, determining whether a patient’s blood oxygen saturation is below a threshold would likely be valuable in an accessible early warning screening tool to indicate that further attention from a clinician is needed. Usability of smartphone screening tools has also been explored but generally shown that accuracy is poor due to the lack of clinical validation and user experience challenges^22–24^.

In this study, we take a step towards SpO_2_ monitoring using the unmodified camera on a smartphone. Our hypothesis was that, by training a model using data from a varied FiO_2_ study, we could accurately predict SpO_2_ on a wider range of clinically relevant SpO_2_ levels (70–100%) than prior smartphone-based studies. Our analysis reveals that a convolutional neural network (CNN) model evaluated on this range is able to achieve, on average, a Mean Absolute Error (MAE) of 5.00% (*σ* = 1.90) SpO_2_ in predicting a new subject’s SpO_2_ level, after it has been trained only on other subjects’ labeled data. To assess potential hypoxemia screening capability, we show that this corresponds to an average sensitivity and specificity of 81% and 79% respectively in classifying a new subject’s SpO_2_ as below 90%. This work builds on a growing tradition of using ubiquitous mobile devices as decision support tools in healthcare, indicating the need for health care consultation^25,26^. Smartphones are widely owned because of their multi-purpose utility, and contain increasingly powerful sensors, including a camera with a LED flash^27–29^. Researchers have used sensors in off-the-shelf smartphone devices to assess many physiological conditions, including detecting voice disorders^30^, tracking pulmonary function^30,31^, assessing infertility^25^, measuring hemoglobin concentration^32,33^, and estimating changes in blood pressure^34,35^. Alongside these results, we share the data from the Varied FiO_2_ study with the community, so others may build on this work.

## Results

### SpO_2_ prediction performance

**Fig. 2:**
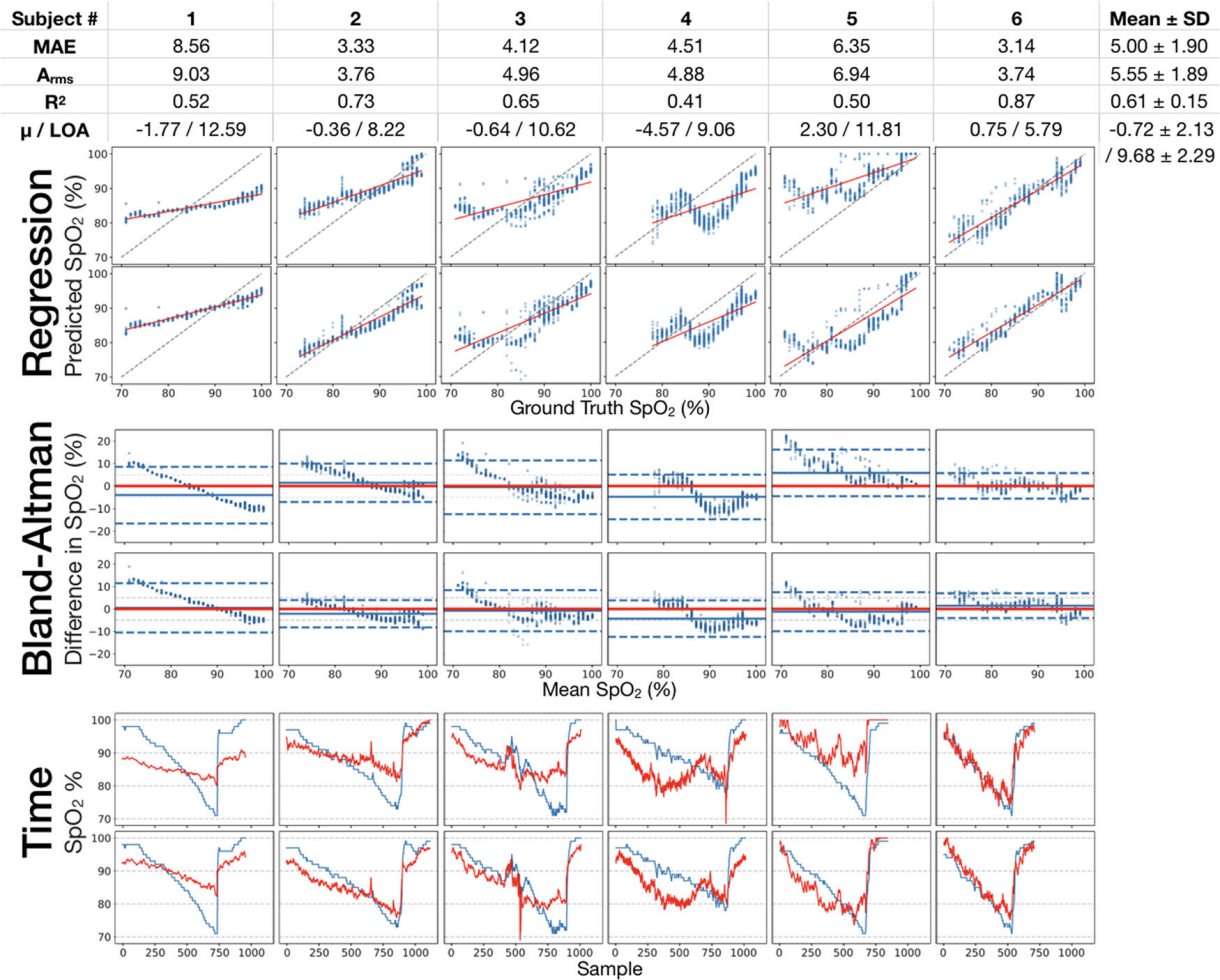
Regression results, Bland-Altman comparison, and time series data from the varied FiO_2_ study. MAE averages to 5.00% (*σ* = 1.90) over all 6 subjects in the study. R^2^ correlation averages to 0.61 over the full range of data gathered. The average difference (*μ*) and limits of agreement (LOA) average to –0.72 and 9.68. Table: MAE and Bland-Altman statistics for CNN evaluation by LOOCV for each subject (*n* = 6) in the study. Regression: Predictions from smartphone data plotted against associated ground truth SpO_2_ data collected via standalone pulse oximeter. Bland-Altman: Bland-Altman plots displaying the spread of predictions against ground truth. Time: Plots of direct performance analysis of regression results. Model predictions (in red) and ground truth readings (in blue) for the 6 subjects in the FiO_2_ study plotted against time of study. For all plots, left hand is on top and right hand is on bottom.

Our convolutional neural network (CNN) achieved an average MAE of 5.00% (*σ* = 1.90) SpO_2_ when trained and evaluated via leave-one-out cross validation (LOOCV) across the range of 70–100% of data from the varied FiO_2_ study (Fig. 2). An average correlation of *R*^2^ = 0.61 (*σ* = 0.15) is observed between the model predictions and readings from the ground truth reference pulse oximeter. The average root mean squared error (A_rms_) is 5.55% (*σ* = 1.89) across all subjects in this range, which is 2.05% higher than the ISO 80601-2-61:2017 standard of 3.5% for reflectance pulse oximeter devices to be cleared for clinical use^13^. Bland-Altman analysis demonstrates the performance of the CNN relative to a tight-tolerance fingerclip pulse oximeter in LOOCV. The SpO_2_ values predicted by the learned model near the Limits of Agreement (LOA) reported in previous studies of clinical and non-clinical pulse oximeters, while evaluating on a wider range of SpO_2_ levels^12,36–38^. Considering that the ground truth measurements from pulse oximeters exhibit similar variance to these results, this indicates that the model has learned features in the PPG signal that are common across subjects and the model is not simply mean-tracking. On the other hand, for Subjects 2, 3, and 5, the negative trend in predictions and mean difference above the limits of agreement for most ground truth values in the range 70–80% SpO_2_ reveals that the model is consistently over-predicting on SpO_2_ samples below 80%. Notably, this is the first study to observe model performance below 85%, as no prior work has demonstrated that smartphone-based sensing systems may perform poorly in this range.

### Classification of hypoxemia

Rather than simply inferring an estimate for SpO_2_, a smartphone-based tool could be valuable for screening for low SpO_2_, indicating whether or not further medical attention is needed. To explore the potential of using an unmodified smartphone camera oximeter system as a screening tool for hypoxemia, we calculated the classification accuracy of our model in providing an indication of whether an individual has an SpO_2_ level below three different thresholds: 92%, 90%, and 88%. A pulse oximetry value below 90% SpO_2_ is a common threshold used to indicate the need for medical attention^39^, but other thresholds could be valuable clinically. Thus, we evaluate the ability of our system to classify samples from our test set by thresholding the regression result from our model at different decision boundaries and comparing it to whether the ground truth pulse oximeter simultaneously reports less than the threshold value. We compute sensitivity (true positive rate) and specificity (true negative rate) across all combinations of LOOCV to compute an average result. This experiment simulates the scenario where a smartphone screens a subject it has never seen before, as the model was trained only on the 5 other subjects from the dataset.

**Fig. 3:**
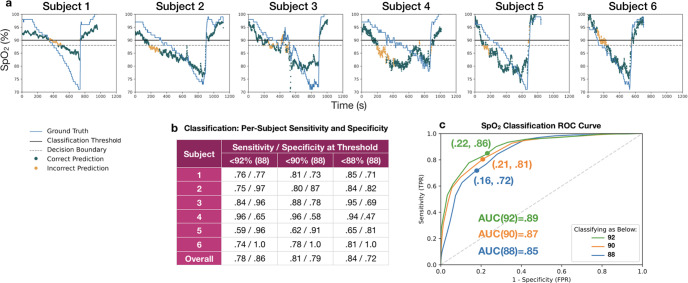
Classification results for the system. **a** Classifications overlaid on ground truth for each subject with a 90% classification threshold and 88% decision boundary. **b** Summary statistics for classification across subjects shows that classification performed better on certain patients, and overall achieved a 81% sensitivity and 79% specificity rate at sensing whether a subject fell below a 90 % SpO_2_ level **c** ROC curves for the classification of low SpO_2_, produced by thresholding the regression model. Classification accuracy decreases as the classification goal is shifted lower, from 92% to 90% to 88%. The classification decision boundary was varied to produce curves for all 3 classification goals, with each point plotted as the average test classification False Positive Rate and True Positive Rate for all LOOCV combinations. The points that are labeled on each curve are those closest to (0,1) for each classification threshold. The Area Under the Curve (AUC) is .87 for the 90% threshold SpO_2_ level classification.

The results of this classification analysis can be seen in (Fig. 3). For classifying SpO_2_ < 90%, on average across all 6 test subjects, our model attains a sensitivity of 81% for correctly classifying the positive samples in our dataset of suspected hypoxemia, while maintaining a specificity of 79%. For classifying a subject as below SpO_2_ < 92%, specificity increases to 86% with a sensitivity of 78%. Not all combinations of test and train subjects displayed the same level of accuracy. In order to visualize classification accuracy across our entire dataset, we varied the classification decision boundary for three classification thresholds that may be clinically relevant, (92%, 90%, and 88%), and averaged the results across all 6 combinations of LOOCV. The results of varying the decision boundary are plotted on the ROC curve in Fig. 3c. For the SpO_2_ < 90% classification threshold, the highest accuracy (defined as the closest point to (0,1) on the ROC curve) occurred when the classification decision boundary was set to 88% SpO_2_. A decision boundary of 90% on the regression result for the SpO_2_ < 90% classification task resulted in 92% sensitivity at identifying hypoxemic cases alongside 35% false positives (sensitivity of 92% and specificity of 65%).

Classification on individual subjects can be seen in Fig. 3a. The model achieved the best performance on Subject 4, with a sensitivity = 88% and specificity = 78%, reporting correctly 88% of the time when the subject had a dangerous SpO_2_ level. Subject 1 displayed the lowest sensitivity to specificity tradeoff of 81% to 73%. As noted in Discussion, the subject had significantly thickened skin on their fingers. Even though the regression for this test subject produces a *MAE* = 8.56%, the classification result indicates that the tool could still be helpful in determining whether or not the user should seek medical attention. Overall, this classification result indicates that the current model is insufficient for medical use, but further research, including collecting a larger data set from a wider array of subjects, may improve the accuracy in the future.

### Data ablation

To understand how the accuracy of our model compares to previously published smartphone-based pulse oximetry systems, we study how excluding subsets of the dataset affects the accuracy. Due to the larger range evaluated in this study compared to prior studies, the overall MAE is not as low as prior studies. However, a data ablation study reveals that, as subsets of the data with lower associated ground truth SpO_2_ readings are removed, the accuracy of our model nears that of other published work. Notably, none of these proof-of-concept works were evaluated on data where a statistically significant portion of the SpO_2_ evaluation data was below 85%, whereas in our varied FiO_2_ dataset, the minimum SpO_2_ value included is 70% and the mean of all ground truth SpO_2_ levels is 87.1% (See Fig. 1c).

**Fig. 4:**
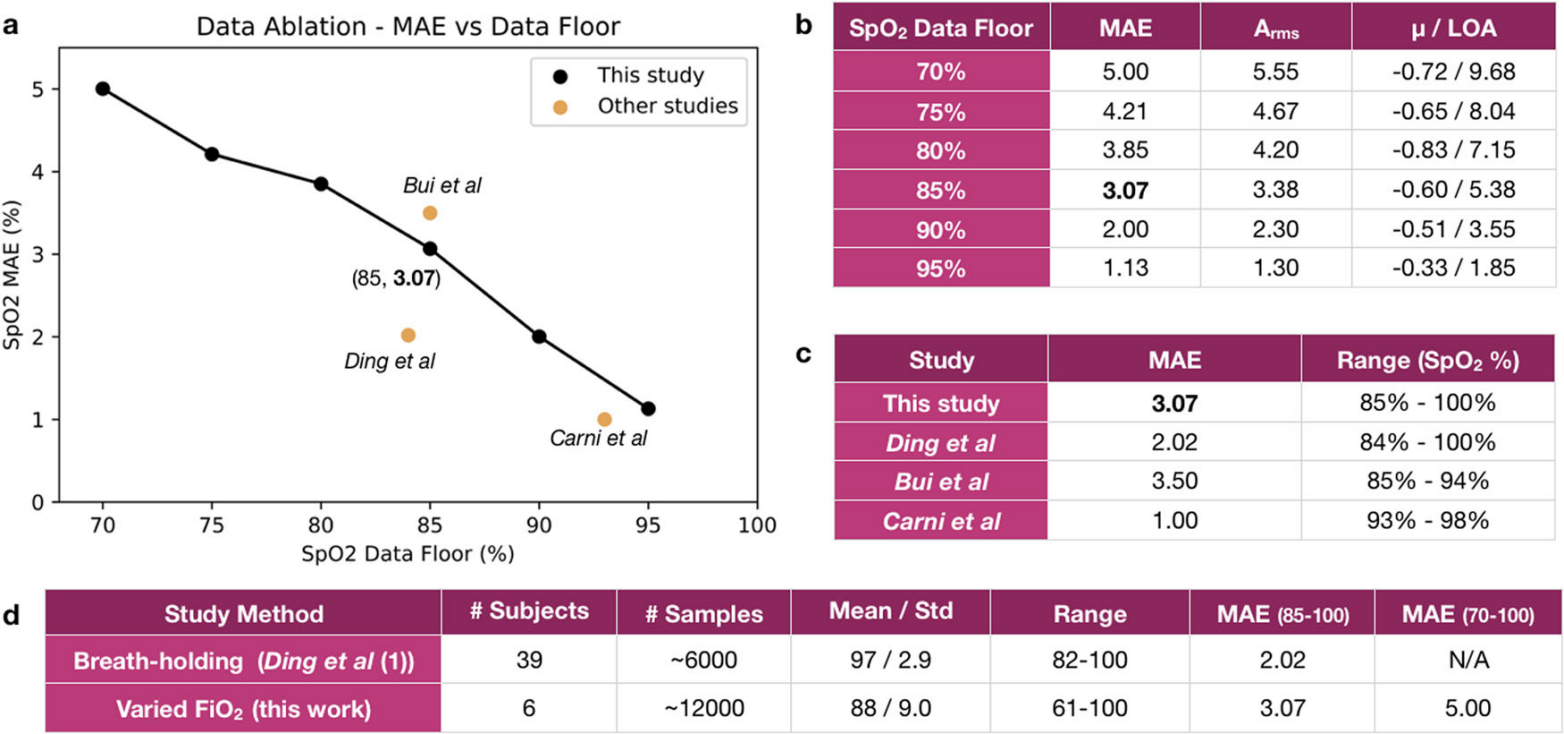
Data ablation study. As shown by a data ablation analysis, our model achieves increased accuracy at smaller ranges of data, such as that in prior studies evaluating above 80% SpO_2_ using a breath-holding study technique. **a** Accuracy of our model improves when ablating our data to remove data below a floor of lowest ground truth SpO_2_ readings. **b** Accuracy statistics from the ablation analysis show that A_rms_ and Limits of Agreement improve with a higher data floor. **c** Mean Absolute Error (MAE) of prior works in smartphone-based SpO_2_ sensing that perform on datasets with SpO_2_ values in the range of 85–100%. When the range of the data in our work is reduced to a similar range, we achieve comparable accuracy to prior work. Note that Bui et al used attachments on the smartphone to enhance the photoplethysmographic signal for inference while Ding et al and the present work use an unmodified smartphone camera^1-3^. **d** Sample statistics and MAE results for this varied FiO_2_ study are compared to a recent breath-holding study using smartphone cameras and deep learning^3^.

We train and evaluate our machine learning models against a similar dataset to these proof-of-concept works using a data ablation technique. We first subsample our dataset so that we only include samples with ground truth SpO_2_ above a floor threshold. We then retrain and evaluate our models to calculate a sub-sampled MAE. Varying across possible thresholds, we observe a negative linear correlation between the minimum SpO_2_ value included and the resultant mean absolute error, as can be seen in Fig. 4a. That is, as we reduce the range of SpO_2_ values in our training and testing dataset, our models perform more accurately. To directly compare to the performance of prior work from Ding et al. and Bui et al. (Fig. 4b), we set a SpO_2_ threshold of 85%. While Ding et al. report a range of 73–100%, their dataset shows that only 0.6% of all samples are below 85% (Fig. 1c), so we report this as a practical floor of 85% for comparison purposes. At a floor SpO_2_ value of 85%, our model performs nearly as well as prior work with a mean absolute error of 3.06%. With this analysis, we can be confident that our techniques are at least as reliable as prior works, and likely benefit from the larger range of training examples.

## Discussion

The classification results from this study indicate a direction to consider for enabling more accessible screening for hypoxemia via unmodified smartphones. Considering the unique positioning of smartphones in the pockets of billions of people worldwide, it would be useful to not only reproduce the function of a pulse oximeter in software, but also to provide an initial screen for clinically significant low SpO_2_ levels. The COVID-19 pandemic highlighted this need for an affordable remote oxygen desaturation detection tool that can be accurately and safely used for initial screening and monitoring, informing users whether or not they should seek expert medical attention. This potential is important to consider, as software applications are already being used in this manner even when those applications have not cleared the FDA regulatory requirements^40,41^. Our system is the first unmodified smartphone camera sensor to report accuracy at levels below 85% SpO_2_, and it achieved relatively high sensitivity (81%) and specificity (79%) when classifying subjects with SpO_2_ below 90%.

**Fig. 5:**
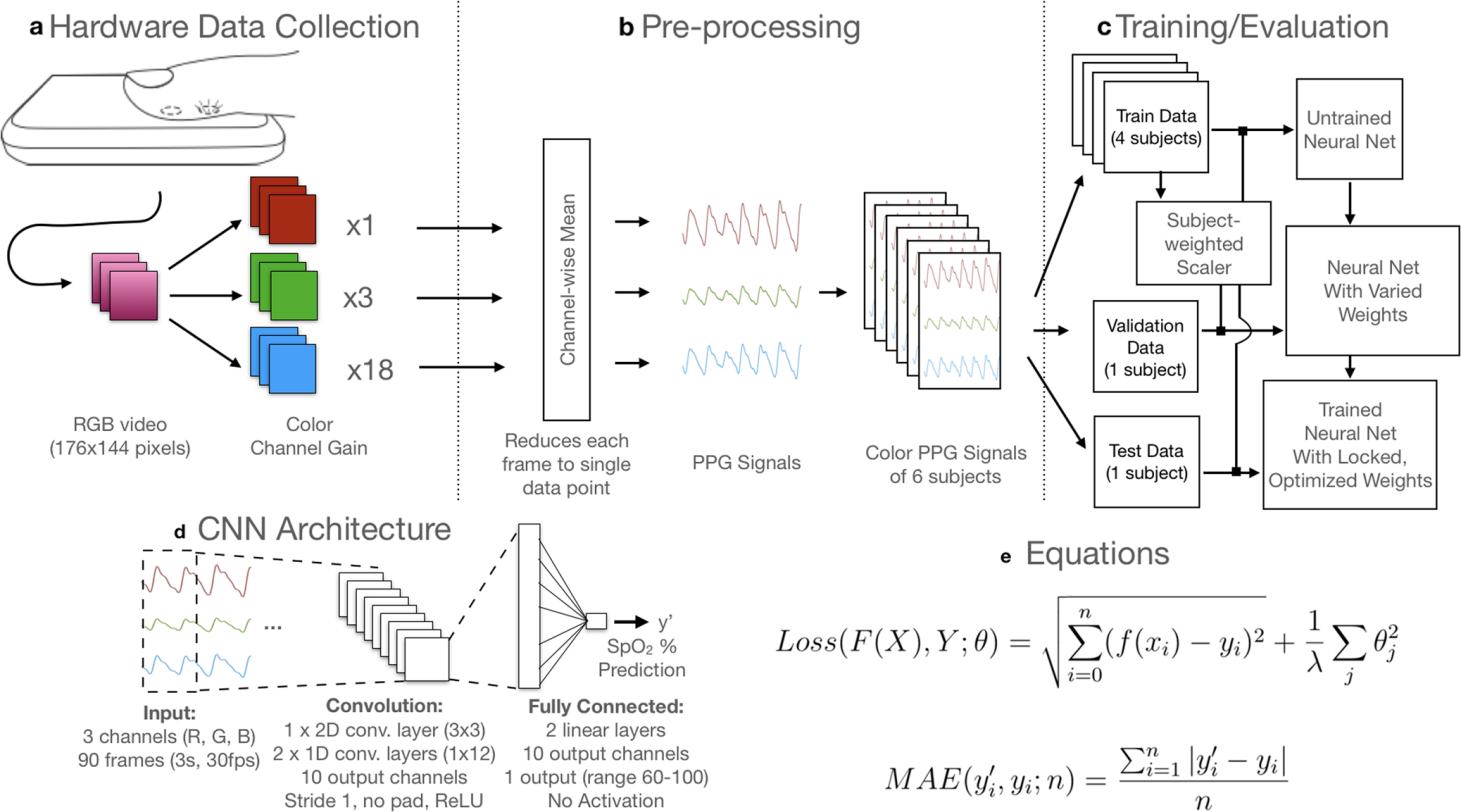
Signal extraction and deep learning pipeline. **a** PPG signal extraction occurs after collecting video data from the smartphone camera, applying empirically determined per-channel gains to ensure that each channel is within a usable range (no clipping or saturating). Gains for the R, G, and B channels were empirically determined and held constant throughout all subjects to avoid clipping or biasing towards one channel. **b** Pre-processing of the data extracts the PPG signal for each channel by computing the average pixel value of each frame. The mean of each channel value across the entirety of each frame was used. **c** Training and evaluation was performed using Leave-One-Out Cross-Validation (LOOCV) by using 5 subjects' data as the training set, holding one of these subject's data as the validation set for optimizing the model, and then evaluating the trained model on one test subject. **d** The deep learning model is constructed of 3 convolutional layers and 2 linear layers operating on the input of 3 s of RGB video data (90 frames for 3s at 30fps). The output is a prediction of the current blood-oxygen saturation (SpO_2_ %) of the individual, which was evaluated using Mean Absolute Error (MAE) compared to the ground truth standalone pulse oximeter reading. **e** Equations for Loss and MAE that were used in training and evaluating the model.

This SpO_2_ prediction pipeline, including smartphone hardware, custom software application, data processing, deep learning and evaluation, is summarized in (Fig. 5). Overall, CNN modeling worked well on this input data, learning a function that approximates the data in a non-linear fashion.

We designed our CNN architecture with three goals in mind. First, we chose the overall number of layers in our model such that there are enough affine computations that the model could learn to approximate the ratio-of-ratios model traditionally used in purpose-built pulse oximeters. Second, we included convolutional layers to provide time-invariance to remain robust to inputs that start at different phases of the heartbeat. Third, the ReLU activation function provided non-linearity, allowing the model to learn features embedded in our wide-band input data, which has more noise than the narrow-band input of purpose-built pulse oximeters traditionally used with a ratio-of-ratios model. Linear layers finally regress from these high-level features to the SpO_2_ prediction.

We investigated other types of models, as well, but were not able to achieve better performance than our CNN model. While a ratio-of-ratios approach^8^ has some predictive power on this full range of smartphone camera data (average MAE = 7.12, *σ* = 1.64), it does not infer SpO_2_ as accurately as our CNN model (Supplementary Fig. 1). This is likely because our CNN model, having more parameters and derived features, handles noise in the signal better than the ratio-of-ratios model. Even so, the fact that the ratio-of-ratios model showed some correlative power (average R^2^ = 0.21, *σ* = 0.20) is encouraging, suggesting that it and the CNN model could have modeled a similar underlying phenomenon. Future work may leverage gradient-weighted activation mappings to further investigate this relationship.

Statistically, our study does not indicate that this smartphone method of measurement and deep learning approach is ready to be used as a medical device comparable with current pulse oximeters, but further studies could be conducted to develop the method and validate for medical use. A Wilcoxon signed-rank test indicates that our observed MAE differences are large enough to reject a null hypothesis that the measures are equivalent with *p* = 0.03, even though the sample size is small (*n* = 6). The ISO standard 80601-2-61:2017 for safety of pulse oximeter devices indicates that at least 10 subjects with diverse skin tones should be tested and result in a root-mean-squared error (A_rms_) below 3.5%, which indicates that more subjects should be tested before we can determine whether this method is accurate enough for clinical use^13,42,43^. In addition, an Arterial Blood Gas (ABG) measurement should be used as ground truth for comparison, and a single model would need to be trained prior to testing on these 10 subjects, rather than the LOOCV procedure that was used in this study.

In addition, we investigated whether heart rate (HR) or respiration rate (RR), which are correlated with acute drops in SpO_2_, were major contributing factors to model accuracy. We found that encoding the input data as 3 beats at 60bpm, effectively removing heart rate as a discernable feature from the input data, only reduced the accuracy of the model by 0.35 to an average MAE = 5.35 (*σ* = 2.20), indicating that HR was not a major contributing factor to model performance (Supplementary Fig. 3). RR was not encoded in the input data, as 3 s is not enough time to see a single breathing cycle for subjects resting in a reclined position. Overall, this level of performance on a relatively small test subject sample (*n* = 6 subjects with s = 12108 total samples) indicates that the model accuracy could increase if more training samples were gathered from further varied FiO_2_ experiments, representing a larger range of potential users of the system.

For this study, camera settings were locked during data gathering by presetting auto-balancing and manually enhancing color gain, which are unique steps in our data collection system relative to prior works in this area. Camera image capture is variably exposed based on three factors: exposure time, sensor sensitivity, and aperture. For RGB cameras used in smartphones, all three color channels typically use the same exposure time and aperture settings. Even though the Bayer filter pattern of CMOS camera sensors is designed to sense twice the green light photons per area, it is sometimes not possible to measure all three channels with high dynamic range simultaneously. Both oxygenated and deoxygenated hemoglobin have a significantly higher absorption coefficient in the blue and green wavelengths than for the red wavelengths by about two orders of magnitude. Thus, it would not be possible to measure all three wavelengths simultaneously under the same exposure. If the hardware sensor’s sensitivity to a particular color is too high or too low, pixel values for that color may clip by recording the minimum or maximum value of 0 or 255. Because phones use an 8-bit precision scheme for storing pixel data, the pixels will all be rounded to 0 and small changes in that color will be lost. In our application, red is the most dominant color, and prior work has shown that with the use of white balance presets for incandescent light, the tones between blue and green can be amplified^44^. Software advancements in smartphone image processing pipelines now provide more independent control of each color channel’s exposure through independent per-channel amplifier gain settings. By having control of independent amplifier gain settings, we can balance the exposure settings to amplify the blue and green channels, as shown in (Fig. 5a).

**Fig. 6:**
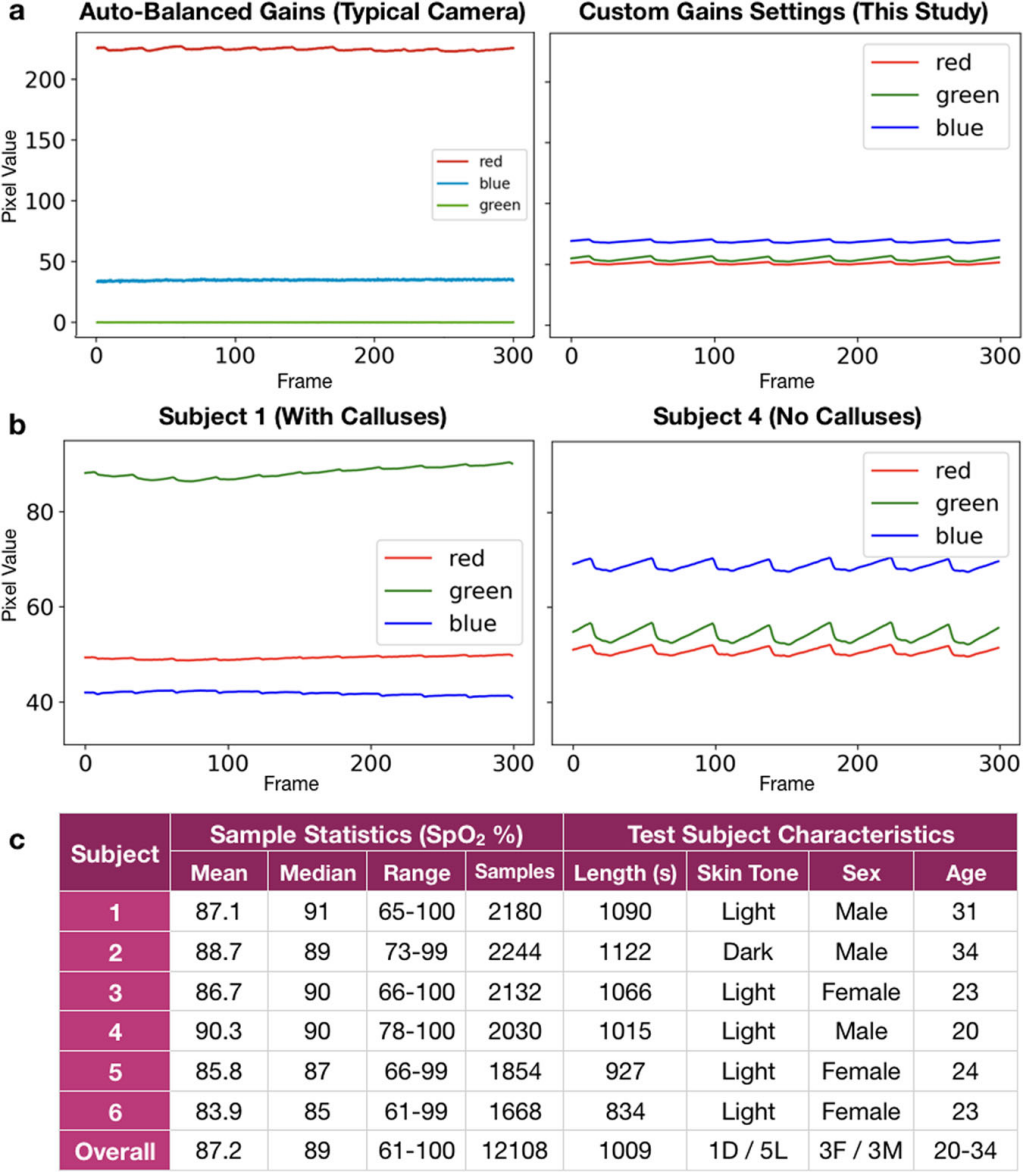
Analysis of collected data. Visualization of PPG data, derived from smartphone videos, reveal the effects of camera gains settings and skin tissue differences on the input signal for our deep learning model. **a** PPG signal using auto-balance from a prior study^3^ vs custom empirically determined gain settings from this study. In the left image, the green channel is clipped so that the dynamic range becomes so low that the AC variation in the signal cannot be observed. In the right image, the pulsation is visible in all three channels. This shows how standard smartphone camera settings, designed for photography, can reduce the information available to smartphone-based systems for accurate SpO_2_ sensing. **b** Skin tissue aberrations (such as calluses seen in Subject 1's fingers) can affect the quality of data available for SpO_2_ sensing. At left, the raw data in the red, blue, and green channels for Subject 1 are dampened and the oscillating portion of the signal cannot be observed at a resolution of 300 frames. At right, the oscillations can be clearly seen for Subject 2 at the same resolution. This abnormality is likely due to Subject 1's callused tissue on the fingers. **c** Subject breakdown for the FiO_2_ study and ground truth data statistics (in SpO_2_ %) for each subject. The average difference between mean and median for each subject is 1.58, showing minimal skew. Skin tone was recorded based on appearance of the skin on the subjects' hand. The average length of each subject's test run is about 16 min.

We see particularly aberrant performance on subject 1 with MAE = 8.56. We suspect this is due to exacerbated tissue noise on the subject’s fingers from thickened skin, which is not represented in the rest of the training data. This subject was noted to be the only subject in the study with noticeable calluses on their fingertips, and the subject indicated this was due to sports. We investigate the data obtained from this subject more closely in (Fig. 6b) and observe that the PPG signals for subject 1 show nearly 50% dampened oscillations (AC signal component) and 50% higher average value (DC signal component) than other subjects. We hypothesize that these abnormal features are a result of the calluses. Specifically, an abnormally thick layer of tissue on the finger would absorb more light in the blue and green spectra. Because our device’s sensor has fixed sensitivity, the abnormally attenuated light in the blue and green spectra results in poor measurement of the pulsatile blood and altered spread in color channel values. With a small training set of 4 subjects including no other examples of subjects with fingertip calluses, the model cannot learn to account for these tissue differences. We anticipate the model could learn to account for tissue abnormalities if trained on more subjects or if adaptive gain settings were employed to gather data that ensured a similar oscillation amplitude in the AC signal for the input data collected by the smartphone.

From this limited dataset, we are unable to make definitive conclusions regarding the effect of skin tone or sex on smartphone pulse oximetry. Our test subjects included 1 subject with a dark skin tone (subject 2 identified as African-American) and 5 subjects with a light skin tone (all other subjects identified as Caucasian), as seen in (Fig. 6c). Our model does not appear to perform differently based on skin tone with this limited dataset, as the results for subject 2 fell in a similar range as other subjects, as seen in (Fig. 2). However, it has been shown that standalone pulse oximeters, such as the one used as the ground truth in our dataset, can produce decreased accuracy on patients with darker skin tones^45,46^. Based on our study, we do not claim any findings around model performance based on skin tone, but that should be evaluated in future studies. Our model also does not perform differently on any subset of our 3:3 female:male sex split. Analyzing performance of our model to users of different skin tones and biological sexes is important, but will require further work to understand.

Our results, in this pilot study of 6 subjects, provide a positive indication that a smartphone could be used to assess risk of hypoxemia without the addition of extra hardware in the future. In order to validate and enable this, we would recommend gathering more data with a smartphone in varied FiO_2_ studies that induce hypoxemia to increase the training data variety and the accuracy of the deep learning model. With an improved model, we could set up user studies in which the app is used in conjunction with a standalone pulse oximeter to measure the accuracy of the software-based solution in real-world scenarios. Usability of the smartphone-based measure could be explored to further enhance the clinical applicability of the findings^22,24^. Further analysis could involve processing or preparing this type of dataset differently, including exploring the use of different ROIs for signal extraction^47^ and gathering the data differently to study the effect of preset camera gains. Additionally, different phone models have different camera sensor configurations and thus the cross-device compatibility of a model should be tested. We would also like to see what others in the community can do with the open-source FiO_2_ data that we are providing alongside this paper. More development and testing could allow this tool to become beneficial for low-cost clinical management of individuals with chronic respiratory conditions, such as COPD, as well as acute respiratory diseases like COVID-19.

## Methods

### Study design

In total, 6 healthy test subjects were recruited and enrolled to participate in a varied FiO_2_ study to evaluate the efficacy of using unmodified smartphone cameras in pulse oximetry. The varied FiO_2_ study was performed using the varied fractional inspired oxygen protocol administered by a clinical validation laboratory, Clinimark, which is a group that performs validation services for medical devices^42^. This experiment was approved by the Institutional Review Board at Clinimark. Written informed consent for each participant was obtained prior to commencing the test procedure. Six subjects were administered controlled fractional mixtures of medical grade oxygen-nitrogen in a controlled hospital setting for 14–19 min. The subjects rested comfortably in a reclined position while the gas mixture was given to induce hypoxemia in a stair-stepped manner. The mixture of oxygen was started at 18–21% and was adjusted downwards in a stair-stepped manner every 1–2 min. The goal was to maintain the subjects’ SpO_2_ level on a plateau for 30 s, with 4 discrete levels within the following ranges: above 93 (subject’s resting SpO_2_ level breathing room air), 89–93, 80–88, 70–79. During this time, the subjects’ fingers were instrumented with multiple transmittance pulse oximeter clips and two smartphone devices, with the smartphone device on the index finger of each hand. In the controlled hospital setting, the ambient light was kept at a constant level of a controlled fluorescent white light and the position of the smartphone did not change between subjects. The ground truth data was recorded using multiple purpose-built pulse oximeters, including a tight-tolerance finger clip pulse oximeter acting as a transfer standard, the Masimo Radical-7, which has a tested A_rms_ of 2% between 70–100% and 3% between 60–80% SpO_2_ and was used as ground truth in this evaluation^48,49^. Variation in ground truth between the tight-tolerance pulse oximeter chosen as a transfer standard (Masimo Radical-7) and other reference pulse oximeters (which were placed on different fingers on different hands) averaged less than 1, measured in the absolute value of the mean difference between all samples, but varied widely in Limits of Agreement, highlighting the differences between approved pulse oximeters (Supplementary Fig. 5). Subject characteristics and data statistics can be seen in (Fig. 6c). Subject observations were recorded, including the observation that one subject, Subject 1 in the analysis, had particularly callused hands. A trained administrator monitored the subjects’ vitals, including SpO_2_, pulse rate, EtCO_2_, respiration rate, ECG rhythm, and FiO_2_, for any abnormalities and would intervene if deemed necessary. The subjects were informed that they could stop the test at any time. The target for minimum SpO_2_ level was 70%, as that is the level above which the ground truth pulse oximeter was validated as accurate, but some subjects’ SpO_2_ level drifted below 70% briefly during testing, and data in that range was excluded from the study (Supplementary Fig. 4).

### Smartphone device configuration and setup

We collected camera oximetry data with a Google Nexus 6P, recording video at 30 frames per second in a custom video capture application developed in Java using Android Studio. The device was specifically configured so that camera exposure settings in the camera hardware did not change throughout the entire study. Color gains were set to 1x for the red channel, 3x for the green channel, and 18x for the blue channel. These gains were chosen empirically by manually analyzing the impact of gain value adjustments on 20 healthy individuals to find gain values that avoided data loss due to compression and obtained optimal signal quality (see Fig. 6a). The Android camera2 API was used to set a target framerate of 30 fps and the phones were plugged in and kept cool with ice packs so the framerate did not dip below 30 fps during the recording. During the varied FiO_2_ study, because the device could overheat from recording continuous video with flash enabled for more than 1 min, we placed clay ice packs around the device to keep its temperature down for the 14–19 min duration of the study. The ice packs were placed strategically to avoid contact with the hand.

### Data pre-processing

For each hand on each subject, we recorded an ordered list of *n* RGB image frames, each with 176 × 144 pixels. To obtain a PPG signal, we computed the mean pixel value for each color channel and obtained a 3 × *n*-shaped matrix of values. Each hand of each subject is treated as a unique subject in the display of results. We divide the data into samples for each 1-second (30 frames) window, combining the 3 s (90 frames) of sample RGB data centered on 1 ground truth SpO_2_ reading as one sample. This provides over 8000 training examples (4 subjects) to our models, with about 2000 samples (1 subject) held out for both the cross-validation and test set for each configuration of LOOCV. Samples under 70% SpO_2_ are removed prior to training and validation, as the samples gathered below 70% were a result of incidental over-shooting of the intended study range of 70–100% and were not represented in every subject.

### Convolutional neural network

We applied a CNN machine learning model, detailed in (Fig. 5). We designed and trained a network with three convolutional layers followed by two fully connected layers. For the first convolution, we treat the RGB channel components of our signals as a second dimension and use kernel sizes of 3 × 3 with no padding. We normalize and standardize both training and validation datasets based on a weighted channel-wise mean and standard deviation of the training dataset, where the weights are scaled by the length each subject’s data collection. A 3 s sample (90 samples at 30 Hz) was chosen as input based on our intuition that it would provide enough input data for the model to see multiple heartbeats for inference, remaining robust to brief sources of noise from movement, while also keeping in mind usability by keeping the length of required recording to a short length. This input choice was validated via hyperparameter search, which optimized model structure, input data, and regularization parameters based on mean cross-validation set loss across all rounds of LOOCV. The model is trained using the Adam optimizer with a learning rate of 0.00001 (with a rate decay by 0.1 after 80 epochs) and an L2 regularization of strength 0.1, using LOOCV with five subjects in the training set (with one held out for cross-validation) and tested on the remaining subject after optimization. We optimize model weights on the cross-validation subject with Mean Squared Error (MSE) as our loss function and report the accuracy of the results by computing the MAE (Fig. 5e) on the test subject. Hyperparameters were selected in a hyperparameter search using the LOOCV method, selecting those parameters based on the lowest average cross-validation MAE prior to final model training. The model is built and trained using the PyTorch library. In addition to testing a CNN model, we attempted to model the data and infer SpO_2_ accurately using a few different models, including a linear regression model and a ratio-of-ratios model. We achieved the highest accuracy when the CNN was applied, so we conducted our analysis on that model, but also report our results from the ratio-of-ratios model in Supplementary Fig. 1.

### Model benchmark

For comparison, we implemented and applied a ratio-of-ratios model^8^ to our data. We apply a variation of the technique in^8^ to each 3 s sample from our data where the PPG signal is stable. To do this, we first segmented each beat and extracted the slopes and heights of systolic peaks. Partial beats on the edge of the sample window were dropped. The average of the slopes and heights from the red and green color channels were used to calculate the SpO_2_ using Eq. (1) from^8^ with the appropriate absorption coefficients. Finally, we fit a linear regression from the calculated SpO_2_ values and ranges of the RGB channels to the ground truth. We analyzed the regression performance of this model, and the results can be seen in Supplementary Fig. 1.

### Statistical analysis

We identified and evaluated two potential usage scenarios for a software-based oximetry solution on a standalone smartphone: (1) as a replacement for traditional pulse oximeters by regressing a continuous SpO_2_ value, and (2) as an at-home screening tool to inform the need for a follow-up with a physician by classifying regression results as below a particular threshold.

We explored the first scenario of pulse oximetry measurement by performing a regression analysis, comparing our smartphone measurement to a purpose-built pulse oximeter with error and Bland-Altman metrics. In our performance assessment, we evaluated models using Leave-One-Subject-Out cross validation (LOOCV). Specifically, we evaluated six validation splits, holding one subject out as the test set in each split (Fig. 5c) and averaging the test MAE for the overall reported MAE (Fig. 2). Signed rank test and skew were computed using the statsmodels library in Python 3.8.

For Bland-Altman analysis, we see minimal skew in our differences with skew = 0.64. Therefore, we calculate Limits of Agreement assuming a normal distribution by computing 1.96 times the standard deviation of the difference between the ground truth and predictions of the validation dataset^50^. We visually examined the ground truth distributions of the splits to ensure there was not a heavy imbalance in the dataset. We experimented with upsampling the minority SpO_2_ range within each training batch. In practice, this increases the weight of mistakes that are made on examples in the minority SpO_2_ range. By weighting the minority SpO_2_ range mistakes, the model should learn features that improve performance on these examples. However, this had little effect on the performance of the model, so most of our experiments were performed without upsampling. We compared the performance of algorithms using Mean Absolute Error and reported A_rms_ and R^2^.

We explored the second scenario of hypoxemia screening by performing a classification analysis, thresholding the ground truth recordings below 3 different SpO_2_ levels (92%, 90%, and 88%) and comparing it to our thresholded regression result. We examined the true positive (sensitivity) and true negative (specificity) rates at different screening decision boundaries (92%, 90%, and 88%) to illustrate the potential performance of the system for use in hypoxemia screening. To interrogate the potential to adjust this decision boundary to bias towards sensitivity or specificity, we varied the decision boundary across the range of 70%–100% and plotted ROC curves for each subject using LOOCV.

### Reporting summary

Further information on research design is available in the Nature Research Reporting Summary linked to this article.

## Supplementary information

Supplemental Material

Reporting Summary

## Data Availability

We provide the data from the varied FiO_2_ study in open source format to the community to allow others to build upon this work. The dataset generated and analysed during the current study are available on Github in the oximetry-phone-cam-data repository: https://github.com/ubicomplab/oximetry-phone-cam-data.

## Code availability

We provide code for pre-processing, visualization, and data-loading that is compatible with the varied FiO_2_ study in open source format to enable the community to more easily start building upon this work. This supporting code is also available on Github in the oximetry-phone-cam-data repository: https://github.com/ubicomplab/oximetry-phone-cam-data.
