# Supplementary material for: Smartphone camera oximetry in an induced hypoxemia study
Source: NPJ Digit Med. 2022 Sep 19;5:146. doi: 10.1038/s41746-022-00665-y (PMC9483471; doi:10.1038/s41746-022-00665-y)
Supplement: Supplementary file 1 — Supplemental Material [file 41746_2022_665_MOESM1_ESM.pdf]

# Supplementary Materials

## Smartphone Camera Oximetry in an Induced Hypoxemia Study

**Authors:** Jason S. Hoffman,[1*†] Varun K. Viswanath,[2,3†] Caiwei Tian,[1] Xinyi Ding,[4] Matthew J. Thompson,[5] Eric C. Larson,[4] Shwetak N. Patel,[1,6] Edward J. Wang[2,3]

**Affiliations:**
[1]Paul G. Allen School of Computer Science and Engineering, University of Washington
[2]Department of Electrical and Computer Engineering, University of California San Diego
[3]The Design Lab, University of California San Diego
[4]Department of Computer Science, Southern Methodist University
[5]Department of Family Medicine, University of Washington
[6]Department of Electrical and Computer Engineering, University of Washington

[*]To whom correspondence should be addressed; E-mail: jasonhof@cs.washington.edu.
[†]These authors contributed equally to this work.

# List of Supplementary Materials

1. **Supplementary Figure 1**: Benchmark Ratio-of Ratios Model Results
2. **Supplementary Figure 2**: Including training data <70% $SpO_2$.
3. **Supplementary Figure 3**: Regression Results after normalizing heart rate
4. **Supplementary Figure 4**: Subject histograms
5. **Supplementary Figure 5**: Ground truth variation
6. **Supplementary Figure 6**: Open source data table
7. **Supplementary Data 1**: Zip file of open source camera oximetry data

# Supplementary Figure 1

| Subject # | 1 | 2 | 3 | 4 | 5 | 6 | Mean ± SD |
|---|---|---|---|---|---|---|---|
| MAE | 10.58 | 6.87 | 7.39 | 9.54 | 6.78 | 7.31 | 7.12 ± 1.64 |
| $A_{rms}$ | 13.28 | 8.52 | 10.58 | 10.92 | 8.49 | 9.41 | 10.20 ± 1.66 |
| $R^2$ | 0.17 | 0.06 | 0.02 | 0.04 | 0.47 | 0.52 | 0.21 ± 0.20 |
| μ / LOA | 6.38 / 22.83 | -0.79 / 16.63 | -5.38 / 17.85 | -9.07 / 11.90 | 2.05 / 16.15 | 3.51 / 17.12 | 1.24 ± 5.16 / 17.08 ± 3.2 |

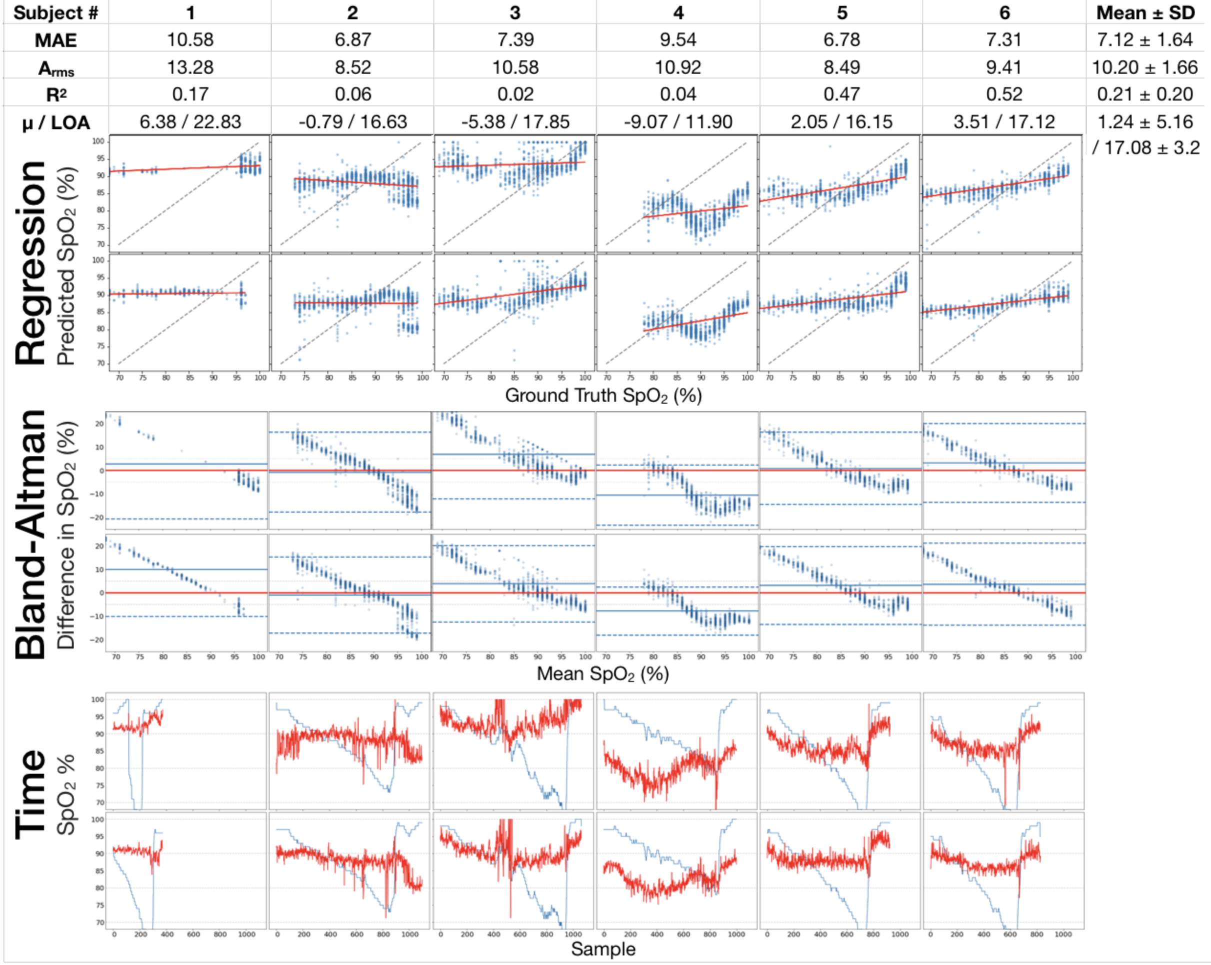


Supplementary Figure 1: **Benchmark Ratio-of-Ratios Model Results.** Applying the ratio-of-ratios model from (*8*) shows that this model does find a pattern in the RGB data gathered from a smartphone, but overall does not perform as well as the CNN model. The average MAE increases by 2.12 to 7.12 ($\sigma$=1.64). On certain portions of the data, such as the portion of Subject 1's data where the peaks are dampened due to calluses attenuating the signal, the slope of the PPG rise falls below 1 and the ratio-of-ratios model is undefined, and thus those samples are dropped from this analysis.

# Supplementary Figure 2

| Subject # | 1 | 2 | 3 | 4 | 5 | 6 | Mean ± SD |
|---|---|---|---|---|---|---|---|
| MAE | 10.52 | 4.07 | 4.42 | 6.01 | 6.88 | 3.09 | 5.83 ± 2.67 |
| $A_{rms}$ | 11.14 | 4.43 | 5.27 | 6.64 | 7.45 | 3.71 | 6.44 ± 2.69 |
| $R^2$ | 0.48 | 0.74 | 0.65 | 0.40 | 0.47 | 0.88 | 0.60 ± 0.19 |
| μ / LOA | -7.15 / 12.55 | -2.80 / 8.24 | -1.09 / 10.10 | -5.40 / 9.38 | 1.92 / 13.03 | -0.17 / 5.62 | -2.45 ± 3.38 / 9.82 ± 2.76 |

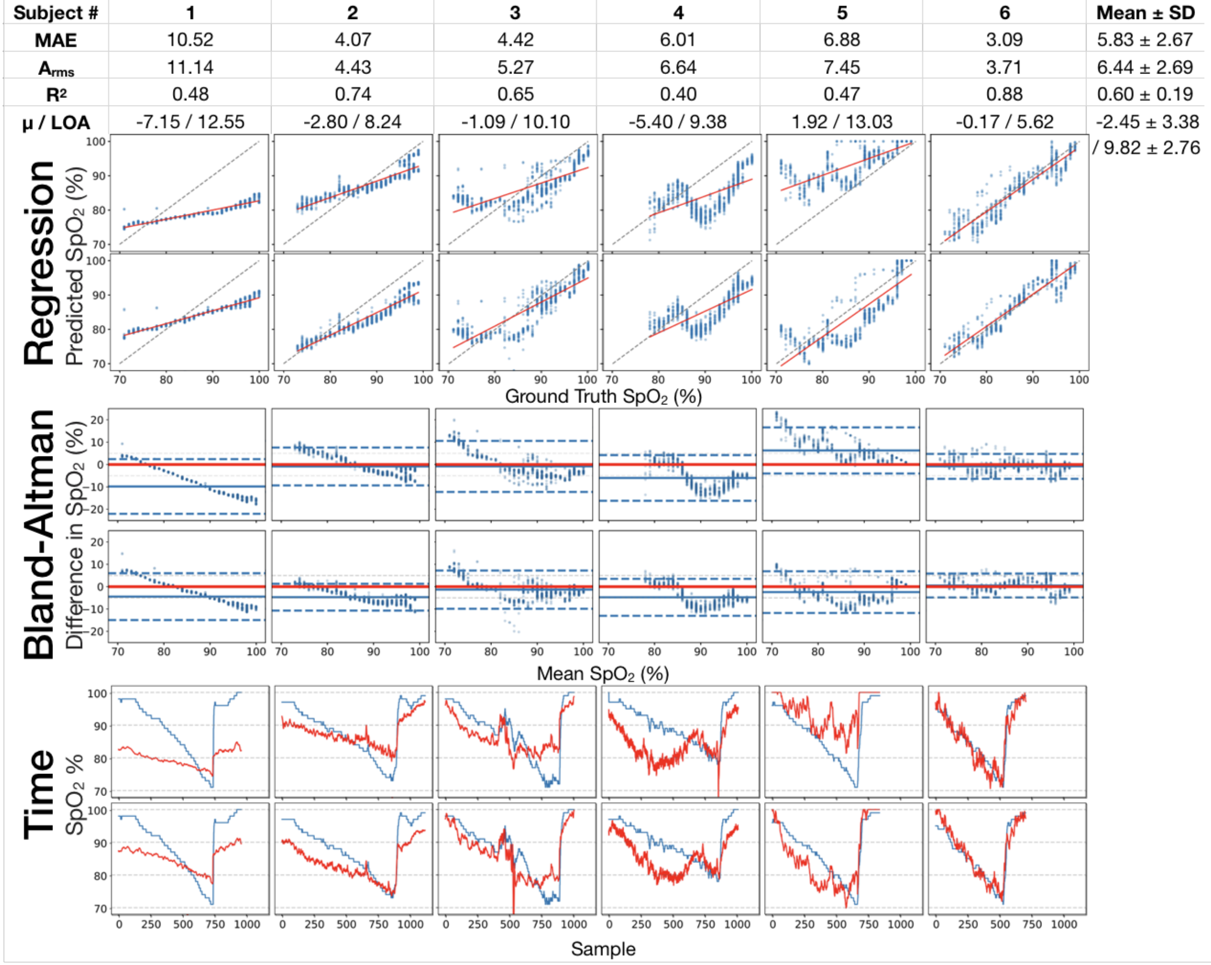


Supplementary Figure 2: **Including training data $<$70% $SpO_2$.** Including all data in training the model, including data below 70% $SpO_2$ ground truth, reduces accuracy of $SpO_2$ inference. This is due to the fact that the ground truth pulse oximeter used as a transfer standard for comparison in this study is not validated to be accurate below 70% $SpO_2$, as it is only required to be validated in the 70% to 100% $SpO_2$ range, as per the ISO standard 80601-2-61:2017 (*13*). Increased error in the training target for training examples below 70% negatively impacts training, reducing the overall accuracy of the resulting model and validity of using the model for inferring $SpO_2$ below 70%. Additionally, the study was designed so that the target for minimum $SpO_2$ level was 70%, and thus, only 2/6 subjects had a significant distribution of data below 70% to train and evaluate. Due to these factors, the analysis of data collected above 70% $SpO_2$ is performed throughout the paper.

# Supplementary Figure 3

| Subject # | 1 | 2 | 3 | 4 | 5 | 6 | Mean ± SD |
|---|---|---|---|---|---|---|---|
| MAE | 9.14 | 3.65 | 4.02 | 5.47 | 6.47 | 3.33 | 5.35 ± 2.01 |
| $A_{rms}$ | 9.69 | 4.03 | 4.92 | 6.00 | 7.00 | 3.86 | 5.92 ± 2.01 |
| $R^2$ | 0.51 | 0.70 | 0.53 | 0.28 | 0.43 | 0.82 | 0.54 ± 0.18 |
| μ / LOA | -3.62 / 12.65 | -1.85 / 7.43 | -0.46 / 10.24 | -5.29 / 8.22 | 2.52 / 12.43 | 0.21 / 5.28 | -1.41 ± 2.55 / 9.37 ± 2.67 |

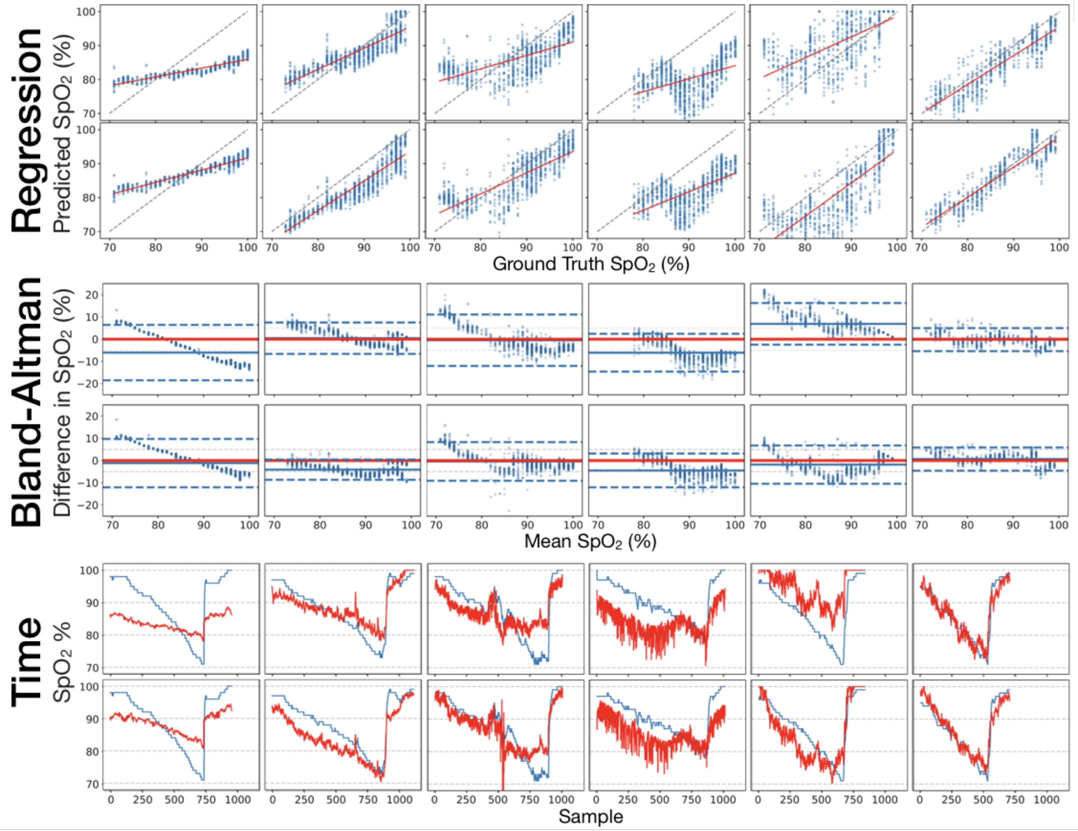


Supplementary Figure 3: **Removing heart rate.** After resampling the data to detect and remove the heartrate, and training the model on 3 beats of 60bpm PPG data, the MAE increases by 0.35 to 5.35 ($\sigma$=1.90) and $R^2$ decreases by 0.07 to 0.54, and additional spread in the predictions is observable for a couple test subjects. This indicates that heart rate may be contributing to the model predictions; however, this cannot explain the full predictive power of the model, as some of this increased error is likely contributed by the HR detection and resampling process.

## Supplementary Figure 4

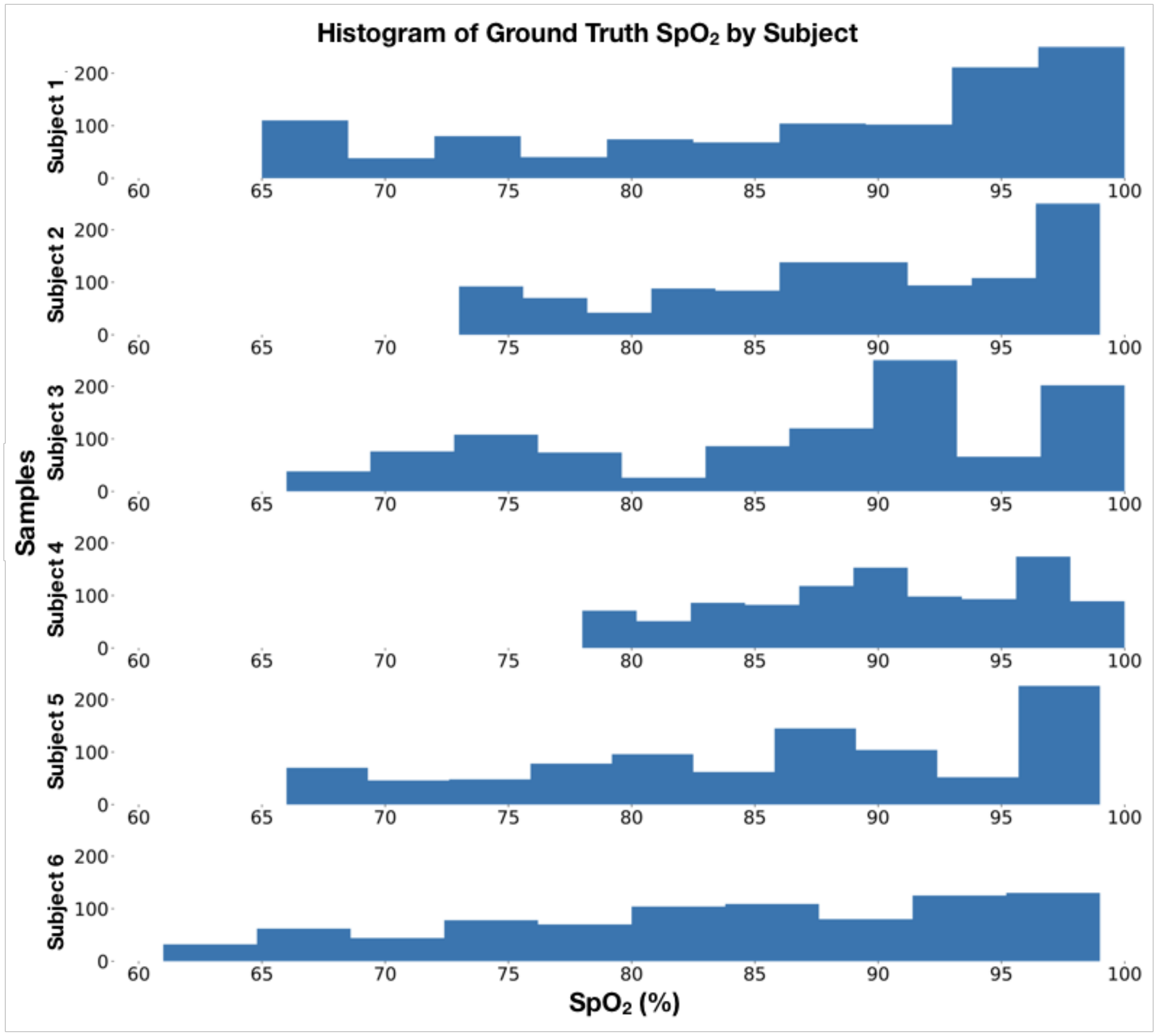


Supplementary Figure 4: **Subject Histograms.** Histograms of ground truth samples gathered from from all 6 test subjects in the study show that all but one subject have a significant spread of data between 70-100%, while only half of the subjects have more than a couple samples below 70%, the minimum $SpO_2$ level defined in the study protocol.

## Supplementary Figure 5

| Ground Truth Pulse Oximeter Device | Difference compared to Masimo Radical 7 | |
|---|---|---|
| | Mean Diff | Std Diff |
| 3900P TT+ | 0.01 | 1.79 |
| Nellcor N-600X 2 | 0.70 | 2.94 |
| Nellcor N-600X 4 | -0.38 | 1.59 |

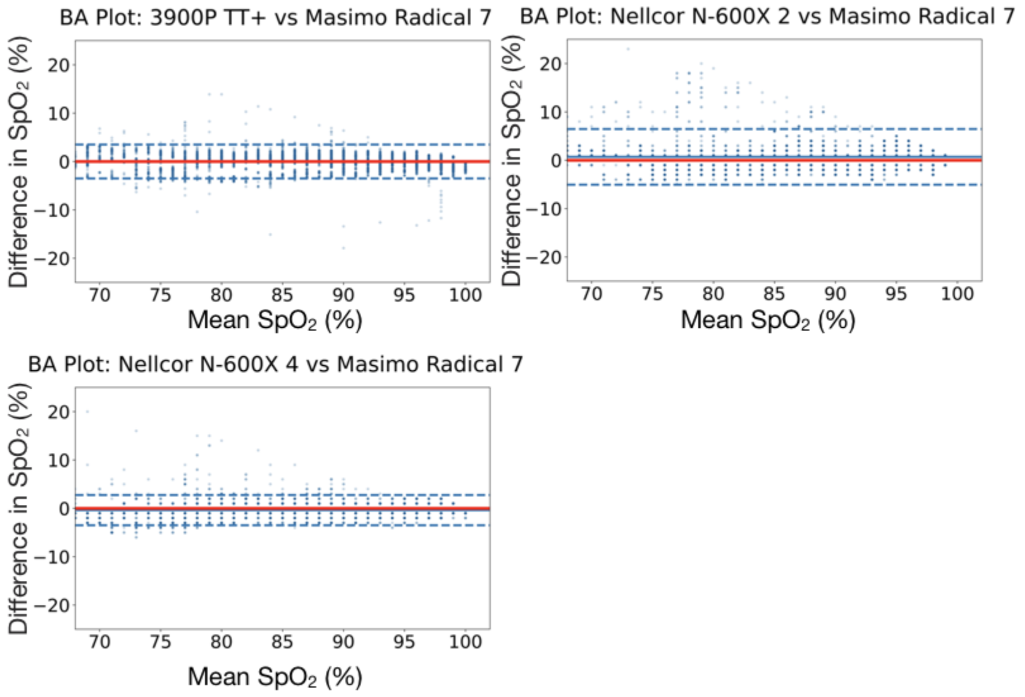


Supplementary Figure 5: **Ground Truth Variation.** Difference plots show that there is some variation in the other pulse oximeters that measured $SpO_2$ level on each subject, compared to the tight tolerance reference pulse oximeter that was used to measure ground truth in this study (Masimo Radical-7). The variation, measured by mean difference was between -0.38 and 0.7, with Standard Difference between 1.59 and 2.94. This variation may have been due to the error range of the pulse oximeters, slight differences in timing between the readings of the pulse oximeters, or differences in value of $SpO_2$ between the subjects' different hands or fingers.

## Supplementary Figure 6

| Subject in Study | Label in Data | | File names | |
|---|---|---|---|---|
| | Num | Label | Left Hand Video<br>Right Hand Video | Info File<br>Ground Truth Data File |
| 1 | 1 | 100001 | 100001-1487003054311-0-1487003073393.mp4<br>100001-1487003016146-0-1487003016393.mp4 | 100001-1487003016146.info<br>100001.csv |
| 2 | 2 | 100002 | 100002-1487006909985-0-1487006910227.mp4<br>100002-1487006911581-0-1487006911849.mp4 | 100002-1487006911581.info<br>100002.csv |
| 3 | 3 | 100003 | 100003-1487010210734-0-1487010211003.mp4<br>100003-1487010212332-0-1487010212594.mp4 | 100003-1487010212332.info<br>100003.csv |
| 4 | 4 | 100004 | 100004-1487016701771-0-1487016702172.mp4<br>100004-1487016703619-0-1487016703979.mp4 | 100004-1487016703619.info<br>100004.csv |
| 5 | 5 | 100005 | 100005-1487019989533-0-1487019989854.mp4<br>100005-1487019992000-0-1487019992346.mp4 | 100005-1487019992000.info<br>100005.csv |
| 6 | 6 | 100006 | 100006-1487023968612-0-1487023968853.mp4<br>100006-1487023971109-0-1487023971496.mp4 | 100006-1487023971109.info<br>100006.csv |

Supplementary Figure 6: **Open Source Data.** Info on filenames in the open source data associated with subject numbers in this study.

**Supplementary Data 1. Zip file of open source camera oximetry data**

A Github repository has been set up with a Zip file of the data collected in this study, documentation, and example data-loading code:

https://github.com/ubicomplab/oximetry-phone-cam-data.
