# Supplementary material for: Smartphone camera oximetry in an induced hypoxemia study
Source: NPJ Digit Med. 2022 Sep 19;5:146. doi: 10.1038/s41746-022-00665-y (PMC9483471; doi:10.1038/s41746-022-00665-y)
Supplement: Supplementary file 2 — Reporting Summary [file 41746_2022_665_MOESM2_ESM.pdf]

nature portfolio

Corresponding author(s): Jason Hoffman

Last updated by author(s): Nov 25, 2021

Reporting Summary

Nature Portfolio wishes to improve the reproducibility of the work that we publish. This form provides structure for consistency and transparency in reporting. For further information on Nature Portfolio policies, see our Editorial Policies and the Editorial Policy Checklist.

Statistics

For all statistical analyses, confirm that the following items are present in the figure legend, table legend, main text, or Methods section.

n/a	Confirmed	
☐	☒	The exact sample size (*n*) for each experimental group/condition, given as a discrete number and unit of measurement
☐	☒	A statement on whether measurements were taken from distinct samples or whether the same sample was measured repeatedly
☒	☐	The statistical test(s) used AND whether they are one- or two-sided<br>*Only common tests should be described solely by name; describe more complex techniques in the Methods section.*
☒	☐	A description of all covariates tested
☐	☒	A description of any assumptions or corrections, such as tests of normality and adjustment for multiple comparisons
☐	☒	A full description of the statistical parameters including central tendency (e.g. means) or other basic estimates (e.g. regression coefficient) AND variation (e.g. standard deviation) or associated estimates of uncertainty (e.g. confidence intervals)
☒	☐	For null hypothesis testing, the test statistic (e.g. *F*, *t*, *r*) with confidence intervals, effect sizes, degrees of freedom and *P* value noted<br>*Give P values as exact values whenever suitable.*
☒	☐	For Bayesian analysis, information on the choice of priors and Markov chain Monte Carlo settings
☒	☐	For hierarchical and complex designs, identification of the appropriate level for tests and full reporting of outcomes
☒	☐	Estimates of effect sizes (e.g. Cohen's *d*, Pearson's *r*), indicating how they were calculated

Our web collection on statistics for biologists contains articles on many of the points above.

Software and code

Policy information about availability of computer code

Data collection	Android, Android Studio
Data analysis	Python 3.8 and Python open source libraries, including pytorch, scipy, sklearn, numpy, matplotlib, pandas, h5py, statsmodels, itertools

For manuscripts utilizing custom algorithms or software that are central to the research but not yet described in published literature, software must be made available to editors and reviewers. We strongly encourage code deposition in a community repository (e.g. GitHub). See the Nature Portfolio guidelines for submitting code & software for further information.

Data

Policy information about availability of data

All manuscripts must include a data availability statement. This statement should provide the following information, where applicable:

- Accession codes, unique identifiers, or web links for publicly available datasets
- A description of any restrictions on data availability
- For clinical datasets or third party data, please ensure that the statement adheres to our policy

We provide the data from the varied FiO2 study in open source format to the community to allow others to build upon this work.

Field-specific reporting

Please select the one below that is the best fit for your research. If you are not sure, read the appropriate sections before making your selection.

☒ Life sciences ☐ Behavioural & social sciences ☐ Ecological, evolutionary & environmental sciences

For a reference copy of the document with all sections, see nature.com/documents/nr-reporting-summary-flat.pdf

Life sciences study design

All studies must disclose on these points even when the disclosure is negative.

Sample size	We used the minimal sample size to prove a proof of concept (n=6 subjects, with >10,000 samples).
Data exclusions	Data below 70% SpO_2 was not intended to be gathered based on the protocol that validates FDA compliance for pulse oximeters and thus was excluded from the analysis.
Replication	We have described our modeling and statistical methods and shared our data so that others may reproduce our work. We believe it will be reproducible.
Randomization	We used the standard validation technique of Leave-One-Out-Cross-Validation (LOOCV), so randomization of subjects was not necessary. We used standard randomization techniques when loading data for training and testing in our deep learning model.
Blinding	We have a small sample size (n=6), so blinding was not possible. We used the standard validation technique of Leave-One-Out-Cross-Validation (LOOCV).

Reporting for specific materials, systems and methods

We require information from authors about some types of materials, experimental systems and methods used in many studies. Here, indicate whether each material, system or method listed is relevant to your study. If you are not sure if a list item applies to your research, read the appropriate section before selecting a response.

Materials & experimental systems

n/a	Involved in the study	
☒	☐	Antibodies
☒	☐	Eukaryotic cell lines
☒	☐	Palaeontology and archaeology
☒	☐	Animals and other organisms
☐	☒	Human research participants
☒	☐	Clinical data
☒	☐	Dual use research of concern

Methods

n/a	Involved in the study	
☒	☐	ChIP-seq
☒	☐	Flow cytometry
☒	☐	MRI-based neuroimaging

Human research participants

Policy information about studies involving human research participants

Population characteristics	The study population will include 5 to 50 healthy non-smoking (or has refrained from smoking for 2 days) competent adults, ages 18 to 50 years. The subject selection will be an equitable distribution of males and females of any race with varying skin tones including subjects with deep pigmentation. The subjects must understand the study and consent to participate by signing the Informed Consent Form. The subjects must be healthy showing no evidence of medical problems as indicated by satisfactorily completing the health assessment form and health screen.
Recruitment	Subjects were recruited via marketing materials from the clinical lab Clinimark, which performs oximeter validation studies on a regular basis.
Ethics oversight	Approved by the Institutional Review Board (IRB) at Clinimark

Note that full information on the approval of the study protocol must also be provided in the manuscript.
